# Expression of serine/threonine protein kinase SGK1F promotes an hepatoblast state in stem cells directed to differentiate into hepatocytes

**DOI:** 10.1371/journal.pone.0218135

**Published:** 2019-06-26

**Authors:** Fouzeyyah Alsaeedi, Rachel Wilson, Charlotte Candlish, Ibrahim Ibrahim, Alistair C. Leitch, Tarek M. Abdelghany, Colin Wilson, Lyle Armstrong, Matthew C. Wright

**Affiliations:** 1 Institute of Cellular Medicine, Newcastle University, Newcastle Upon Tyne, United Kingdom; 2 Faculty of Medical Sciences, Taif University, Taif, KSA; 3 Institute Human Genetics, Newcastle University, Newcastle Upon Tyne, United Kingdom; 4 Freeman Hospital, Newcastle Upon Tyne, United Kingdom; 5 Department of Pharmacology and Toxicology, Faculty of Pharmacy, Cairo University, Cairo, Egypt; University of Tsukuba, JAPAN

## Abstract

The rat pancreatic AR42J-B13 (B-13) cell line differentiates into non-replicative hepatocyte-like (B-13/H) cells in response to glucocorticoid. Since this response is dependent on an induction of serine/threonine protein kinase 1 (SGK1), this may suggest that a general pivotal role for SGK1 in hepatocyte maturation. To test this hypothesis, the effects of expressing adenoviral-encoded flag tagged human SGK1F (AdV-SGK1F) was examined at 3 stages of human induced pluripotent stem cell (iPSC) differentiation to hepatocytes. B-13 cells infected with AdV-SGK1F in the absence of glucocorticoid resulted in expression of flag tagged SGK1F protein; increases in β-catenin phosphorylation; decreases in Tcf/Lef transcriptional activity; expression of hepatocyte marker genes and conversion of B-13 cells to a cell phenotype near-similar to B-13/H cells. Given this demonstration of functionality, iPSCs directed to differentiate towards hepatocyte-like cells using a standard protocol of chemical inhibitors and mixtures of growth factors were additionally infected with AdV-SGK1F, either at an early time point during differentiation to endoderm; during endoderm differentiation to anterior definitive endoderm and hepatoblasts and once converted to hepatocyte-like cells. SGK1F expression had no effect on differentiation to endoderm, likely due to low levels of expression. However, expression of SGK1F in both iPSCs-derived endoderm and hepatocyte-like cells both resulted in promotion of cells to an hepatoblast phenotype. These data demonstrate that SGK1 expression promotes an hepatoblast phenotype rather than maturation of human iPSC towards a mature hepatocyte phenotype and suggest a transient role for Sgk1 in promoting an hepatoblast state in B-13 trans-differentiation to B-13/H cells.

## Introduction

A common difficulty encountered with stem cell-derived differentiated cells in vitro, is their maturation into fully differentiated phenotypes that quantitatively reflect the function of cells in vivo or directly after isolation from tissues [1–3]. The main functional cell of the liver–hepatocytes–is an exemplar of this problem [4]. Hepatocytes in vivo are highly metabolically active and perform a diverse range of functions (many of which are specific to this cell type). Stem cell-derived hepatocyte-like cells resist functioning as hepatocytes in vitro likely because of several drivers. These include sub-optimal differentiation protocols; a sub-optimal in vitro environment (e.g. extracellular matrix, appropriate cell-cell contacts, cell density) and aberrant levels of regulating factors (e.g. hormones controlling gene expression). These in combination, promote a de-differentiation process, a response also encountered when hepatocytes are isolated from intact organs and placed under similar conditions in vitro [4]. These issues have resulted in extensive efforts to manipulate the in vitro environment to generate and/or preserve hepatic functionality (e.g. co-culture systems [5], 3D culture systems [6], flow cultures [7] etc.).

Liver disease models and stem cell-derived hepatocyte-like cell engraftment studies suggest that stem/progenitor cell-derived cells retain the capacity to function sufficiently as hepatocytes (when in the appropriate in vivo environment) [8–10]. Accordingly, the extent to which stem cell-derived hepatocytes will find general usage for in vitro studies (e.g. drug metabolism and toxicity studies), will depend on how complicated and expensive it will be to replicate the in vivo environment in culture systems.

An alternative approach to generating more mature phenotypes in vitro is through forced over-expression of appropriate transcription factors. The AR42J-B13 (B-13) cell gives credence to this scenario since B-13 cells are able to adopt a mature hepatocyte phenotype (B-13/H cells) in the absence of a complicated culture environment.

B-13 cells are proliferative rat cells expressing a limited set of genes associated with pancreatic acinar cells [11]. In response to glucocorticoids, B-13 cells replicatively senesce, alter morphologically and express many of the genes enriched, or specific to hepatocytes, at levels quantitatively similar to normal rat hepatocytes [12–14,11]. The mechanism under-pinning this differentiation involves an activation of the glucocorticoid receptor; critical epigenetic alterations; induction of serine/threonine protein kinase 1 (Sgk1); Sgk1-dependent repression of constitutive WNT cell signaling activity and expression of a host of transcription factors that drive an hepatic phenotype [14–16]. This response occurs on simple plastic substrata (though it may also occur in 3D and is enhanced by extracellular matrix [17]) and–in contrast to normal hepatocytes–is not reversed by de-differentiation, at least for several weeks [14].

SGK1 is transcriptionally upregulated by glucocorticoids and mineralocorticoids and it primarily regulates epithelial Na^+^ channel (ENaC) and sodium re-absorption by the kidney [18]. SGK1 has been shown to play roles in cell differentiation [19,20] however, these are likely to be redundant since there is no apparent phenotype–developmental or otherwise—in Sgk1^-/-^ mice (although there is impaired renal Na^+^ retention if mice are exposed to salt depletion [21,22]). Since selected isoforms (see methods section for details) of Sgk1 have been shown to substitute for glucocorticoid and be critical for promoting differentiation to B-13/H cells [15], we hypothesized that expression of the human SGK1F isoform will promote and maintain an hepatocyte phenotype in human stem cells directed to differentiate into hepatocyte-like cells. We demonstrate that an AdV-encoded SGK1F capable of efficiently promoting an hepatocyte-like phenotype in B-13 cells promoted hepatoblast formation when SGK1F was expressed in both iPSC-derived endoderm and hepatocyte-like cells.

## Materials and methods

### Materials

Wnt signalling reporter constructs “Topflash” and “Fopflash” were obtained from Addgene and used as previously described [14] Human liver tissue for hepatocyte isolation was ethically obtained via the Newcastle Biobank (https://www.ncl.ac.uk/biobanks/) with over-arching ethical approval from the Newcastle & North Tyneside 1 Research Ethics Committee.

AdV-GFP was generously provided by Dr Audrey Brown (Newcastle University). The pFLAG-CMV2 SGK1F construct encoding 2–241 amino acids of the flagged protein (Wallace et al., 2011) was sub-cloned into adenovirus to produce a replication incompetent AdV-SGK1F by Vector Biolabs (Eagleville, PA). SGK1F was chosen for these studies because there is no orthologue for this variant in rat and therefore ectopic expression and any associated effects could be readily monitored.

### Cell culture

B-13 cells were routinely cultured in low glucose (1g/L) Dulbecco’s Modified Eagle’s Medium (DMEM) containing 10% (v/v) fetal calf serum (FCS), 100units/ml penicillin, 100μg/ml streptomycin and 0.584g/L L-glutamine. B-13 cells were differentiated into B-13/H cells by treatment with dexamethasone (DEX) through addition of 1000-fold molar ethanol solvated stocks, with controls treated with 0.1% (v/v) ethanol vehicle, as previously described [14].

Human iPSCs were generated from dermal fibroblast (Lonza) using a CytoTune‐iPS 2.0 Sendai reprogramming kit (Invitrogen) as described [23,24]. Cells were differentiated into hepatocyte-like cells essentially as described by [25]. In brief, stem cells for conversion into hepatocyte-like cells were routinely sub-cultured into 6 well plates in CDM-PVA media (1:1 v/v mix of DMEM-F12 medium and Iscove's Modified Dulbecco's Medium [Gibco 21980] containing 1g/L polyvinyl alcohol, lipids [Life technologies cat#11905031], thioglycerol, insulin, transferrin and100units/ml penicillin, 100μg/ml streptomycin [25]) with the addition of 100ng/mL activin A and 100ng/mL basic fibroblast growth factor (FGF2). After 24 hours, the cells were washed with calcium-and magnesium-free PBS (137 mM NaCl, 27 mM KCl, 100 mM phosphate pH 7.4) and treated with freshly prepared CDM-PVA media containing 100ng/mL activin-A, 100ng/mL FGF2, 10ng/mL bone morphogenetic protein 4 (BMP4), 10μM LY294002 and 3μM CHIR99021 for 24 hours, followed by freshly prepared CDM-PVA with 100ng/mL activin-A, 100ng FGF2, 10ng/mL BMP-4 and 10μM LY294002 for 24 hours to produce a culture enriched for definitive endodermal cells. The culture medium was then changed to RPMI-B27 medium containing 100ng/mL activin-A and 100ng/mL FGF2 to promote the formation of anterior definitive endodermal cells. From day five to seven days, cells were treated daily with RPMI-B27 differentiation medium with Activin-A (50 ng/ml). At this time, ADE specification takes place. At day 8, hepatic differentiation was induced by replacing medium with RPMI-B27 differentiation medium containing 20ng/mL BMP4 and 10ng/mL fibroblast growth factor (FGF10). The medium was changed daily for the following 4 days leading to the cell cultures enriched for hepatoblasts. Formation of hepatocyte-like cells was subsequently promoted by culturing hepatoblasts in basal hepatocyte medium (Lonza CC-3199) containing 30ng/mL oncostatin-M (OSM) and 50ng/mL hepatocyte growth factor (HGF). The medium was changed every 2 days.

Human hepatocytes were isolated from liver tissue after use in previous research transplant projects associated with organ re-conditioning by collagenase perfusion essentially as previously described [26].

All cells were cultured in an humidified incubator at 37°C and an atmosphere of 5% CO_2_ in air.

### Adenoviral production, titre and MOI determinations

Replication-deficient adenovirus were produced via HEK293 (Human embryonic kidney 293) cells routinely seeded into six-well plates at 1 x 10^5^ cells/well and cultured in low glucose (1g /L) DMEM containing 10% (v/v) FCS, 100units/ml penicillin, 100μg/ml streptomycin and 0.584g/L L-glutamine until approximately 80% confluent. Viral stocks were serially diluted typically between 10^−4^–10^−9^. The medium was removed from the HEK293 cells and 300μl of each adenoviral dilution added to each well, and the plate gently rocked to ensure coverage of all cells by the viral solution and returned to the incubator for 1 hour. During this incubation, a 5% agarose solution in PBS was melted and cooled to 45°C separately with culture medium. After the 1 hour incubation for infection, the viral dilutions were aspirated from the cells, 5% agarose solution was diluted to 0.5% in culture medium and 2ml of this solution was added per well and left to set. Once set, a further 2ml of culture medium was added per well and the cells typically cultured for 7–10 days. Medium was then removed and cells were stained with 0.03% (w/v) neutral red in PBS for 2 hours. The viral titre was determined from the number of plaques counted.

For the determination of MOI in any particular cell type, typically cells were expanded to approximately 80% confluence and infected with a serially-diluted range of titred AdV-GFP. Infected cells were then returned to the incubator. Cultures were examined daily using a fluorescent microscope for up to 4 days and the percentage of cells positive for green fluorescent protein (GFP) determined from 5 randomly selected fields. The titre and approximate number of cells was used to estimate the multiplicity of infection (MOI).

For examining the effects of SGK1 expression, a day prior to infection, cells were sub-cultured into 6 well plates to give approximately 50% confluency in 6 well plates the next day. Cells were then infected with either AdV-SGK1F or AdV-null using an MOI based on the number of cells seeded. Uninfected cells were included as controls. The cells were in some case re-infected–with MOI altered as necessary–based on the extent of any apparent toxicity to previous infections in order to optimise both expression and cell viability. Limited trial and error suggested that that this approach gave the best infection rates whilst limiting losses in cell viability.

### SGK1 activity

SGK1 kinase activities were determined using an ADP-Glo Kinase Assay (Promega, UK). In brief, cells were harvested and snap frozen in liquid nitrogen whilst protein contents determined. Defrosted cell lysates were diluted to 500 ng protein/ml and the assay performed essentially according to the manufacturer’s instructions using the AKT substrate provided. After the SGK1 kinase reaction was completed, an equal volume of ADP-Glo Reagent was added and incubated for 40 minutes to terminate the kinase reaction and deplete remaining ATP. The ADP produced from this reaction was then converted to ATP, the levels of which were determined using a luciferase/luciferin reaction and an ADP (also converted to ATP) calibration curve.

### Fluorescence immunocytochemistry

Cells in 6 well plates were washed twice with PBS then permeabilised with 2ml of ice-cold absolute methanol at 4°C for 10 minutes. The methanol was then aspirated and the cells washed twice with PBS before fixation in 2ml of 0.2% (v/v) glutaraldehyde and 2% (v/v) formaldehyde in PBS, pH 7.4. After 15 minutes at room temperature, the cells were incubated with 5% (v/v) FCS in PBS for 10 minutes to block non-specific binding of antibodies. After washing twice in 10mLs of PBS, the cells were incubated with a 1:200 dilution of a primary antibody (rabbit anti-CpsI [Abcam #3682]; rabbit anti-Cyp2e1 [Abcam #28146]; mouse anti-Hnf4α [Abcam #41898]; mouse anti-Pdx1 [DHSB #F6A11] and either a mouse anti-SGK1 [Santa Cruz sc377360] or rabbit anti-SGK1 [Sigma S5188] dependent on the species of the co-staining antibody) for 1 hour followed by extensive washing in PBS followed by incubation with the appropriate 1:200 FITC- and TRITC-conjugated anti IgGs. Replicate wells stained identically except for incubation with primary antibody were routinely included to determine background level of fluorescence (no 1^o^ Ab control). After extensive washing in PBS, the cells were incubated with the DNA intercalator 4',6-diamidino-2-phenylindole (DAPI, 6μg/ml in PBS) for 20 minutes to identify cell nuclei. The cells were washed three times with PBS before and stored in 5ml of PBS at 4°C in the dark before visualisation.

### Western blotting

Cell proteins were subjected to Western blotting as previously described (Wallace et al., 2010b). Nitrocellulose membranes were probed for targets proteins using 1:1000–1:3000 dilutions of the appropriate primary antibodies (Anti-β-catenin [#Ab 6302]; anti-β-catenin phosphorylated at serine 33 and 37 [# Ab 11350]; rabbit anti-CPSI [Abcam #3682]; rabbit anti-albumin [Sigma, A6154]; rabbit anti-CYP2E1 [Abcam # A28146]; rabbit anti-SGK1 [Cell Signalling #3272]; mouse anti-β-actin [Sigma A54410]) followed by incubation with a 1:3000 dilution of the appropriate species-specific horseradish peroxidase-conjugated secondary antibody (HRP-conjugated goat anti-rabbit IgG [Sigma A6154]; HRP-conjugated goat anti-mouse IgG [Dako P0447]) and visualisation using enhanced chemical luminescence (ECL, Thermo-Scientific).

### Transfection and reporter gene expression

All plasmid constructs were propagated in Top10 cells (Invitrogen, Paisley, UK) and purified using miniprep and maxiprep purification kits (Qiagen, Southampton, UK) following the manufacturer’s protocols. Prior to transfection with plasmids, B-13 cells were sub-cultured into 24 well plates and were transfected (0.34μg DNA/well) from a stock plasmid mixture with a fixed 6:1 ratio of a luciferase reporter and RL-TK (Promega) constructs (to control fro transfection efficiencies between wells). Cells were harvested up to 72 hours after transfection and reporter gene expression determined using a Dual Luciferase Reporter Assay kit (Promega) following the manufacturer’s instructions.

### RT-PCR and qRT-PCR

Medium was aspirated and cells were washed in PBS prior to isolation of total RNA using TRIzol (Invitrogen) according to the manufacturer’s protocol. For qRT-PCR, High Pure RNA Isolation Kits were used (Roche) according to the suppliers instructions and which included treatment of RNA with DNase. RNA was finally re-suspended in RNase-free water, quantified by UV spectrophotometry using a nanodrop 2000 Thermo Scientific) before being aliquoted and stored at -80°C until needed. RNA samples were routinely diluted to 200ng/μl and 5ng/μl for RT-PCR and qRT-PCR respectively. First strand cDNA synthesis and PCR steps were carried out essentially as previously described [15] using M-MLV (Promega). Go-Taq green master mix (Promega) was used to amplify DNA for RT-PCR. For primer sequences and annealing conditions for SGK1F, Cyp2e1, CpsI and Gapdh used for rat samples, see [27,16]. For other primer sequences not previously published, see Table 1.

**Table 1:** Primers used for RT-PCR.

Oligo ID	Primer sequence (5'-3')		Comments
**hSOX2**	**US**	TTCATCGACGAGGCTAAGCG	Will hybridise to human SOX2 cDNA sequence [NM_003106] and amplify a fragment of 255bp.
	**DS**	CATCATGCTGTAGCTGCCGT	
**hSOX17**	**US**	CAAGGGCGAGTCCCGTATC	Will hybridise to human SOX17 cDNA sequence [NM_022454] and amplify a fragment of 132bp.
	**DS**	CACGACTTGCCCAGCATCTTG	
**hFOXA2**	**US**	ACTGTTTCCTGAAGGTGCCC	Will hybridise to human FOXA2 cDNA sequence transcript variant 1[NM_021784] and transcript variant 2 [NM_153675] to amplify a fragment of 224bp.
	**DS**	CTCCCCGAGTTGAGCCTGTG	
**hHHEX**	**US**	CCCTGGGCAAACCTCTACTC	Will hybridise to human HHEX cDNA sequence [NM_002729] and amplify a fragment of 227bp.
	**DS**	TCTCCTCCATTTAGCGCGTC	
**hAFP**	**US**	TGCAGCCAAAGTGAAGAGGGAAGA	Will amplify sequence of 217bp (Kamiyama et al., 2006).
	**DS**	CATAGCGAGCAGCCCAAAGAAGAA	
**hCYP3A7**	**US**	TTCACAAACCGGAGGCCTTT	Will hybridise to human CYP3A7 cDNA sequence [NM_000765] and amplify a fragment of 362bp.
	**DS**	GAGAGAACGAATGGATCTAATGGA	
**hAlbumin**	**US**	CTTGAATGTGCTGATGACAGG	Will amplify sequence of 157bp (Sirico et al., 2012).
	**DS**	GCAAGTCAGCAGGCATCTCAT	
**hCYP3A4**	**US**	CTTCATCCAATGGACTGCATAAAT	Will amplify sequence of 87bp (Bowen et al., 2000).
	**DS**	TCCCAAGTATAACACTCTACACAGACAA	
**hGAPDH**	**US**	TGACATCAAGAAGGTGGTGAAG	Will hybridise to human GAPDH cDNA sequence [NM_001256799, NM_001289745, NM_001289746, NM_001357943 and NM_002046] and amplify fragments all of 180bp.
	**DS**	TTGTCATACCAGGAAATGAGCT	

For qRT-PCR, TaqMan Universal PCR Master Mix and a gene-specific primer and probe mixture (pre-developed TaqMan Gene Expression Assays) were used to target the following human transcripts (OCT4, Hs04260367_gH; SOX2, Hs01053049_s1; HHEX, Hs00242160_m1; FOXA2, Hs00232764_m1; HNF4α, Hs00230853_m1; AFP, Hs01040598_m1; CYP1A2, Hs00167927_m1; CYP3A4, Hs00604506_m1; CYP3A7, Hs02511627_s1; CPS1, Hs00157048_m1; Albumin, Hs00609411_m1) and DNA amplified using an Applied Biosystems 7500 fast thermocycler. Transcript levels between treatments were determined using the comparative ΔΔCt method by normalising to 18S ribosomal RNA (18S rRNA).

### Statistical analysis

For the comparison between two groups, an unpaired Students t-test was carried out and significance assumed where p < 0.05. For comparison of multiple groups, ANOVA was carried out and where significant, differences between groups were determined using Tukey post hoc test. Where p<0.05, a significant difference was assumed.

## Results

### Adenoviral-mediated expression of SGK1F alone induces the conversion of B-13 cells into B-13/H cells

**Fig 1:**
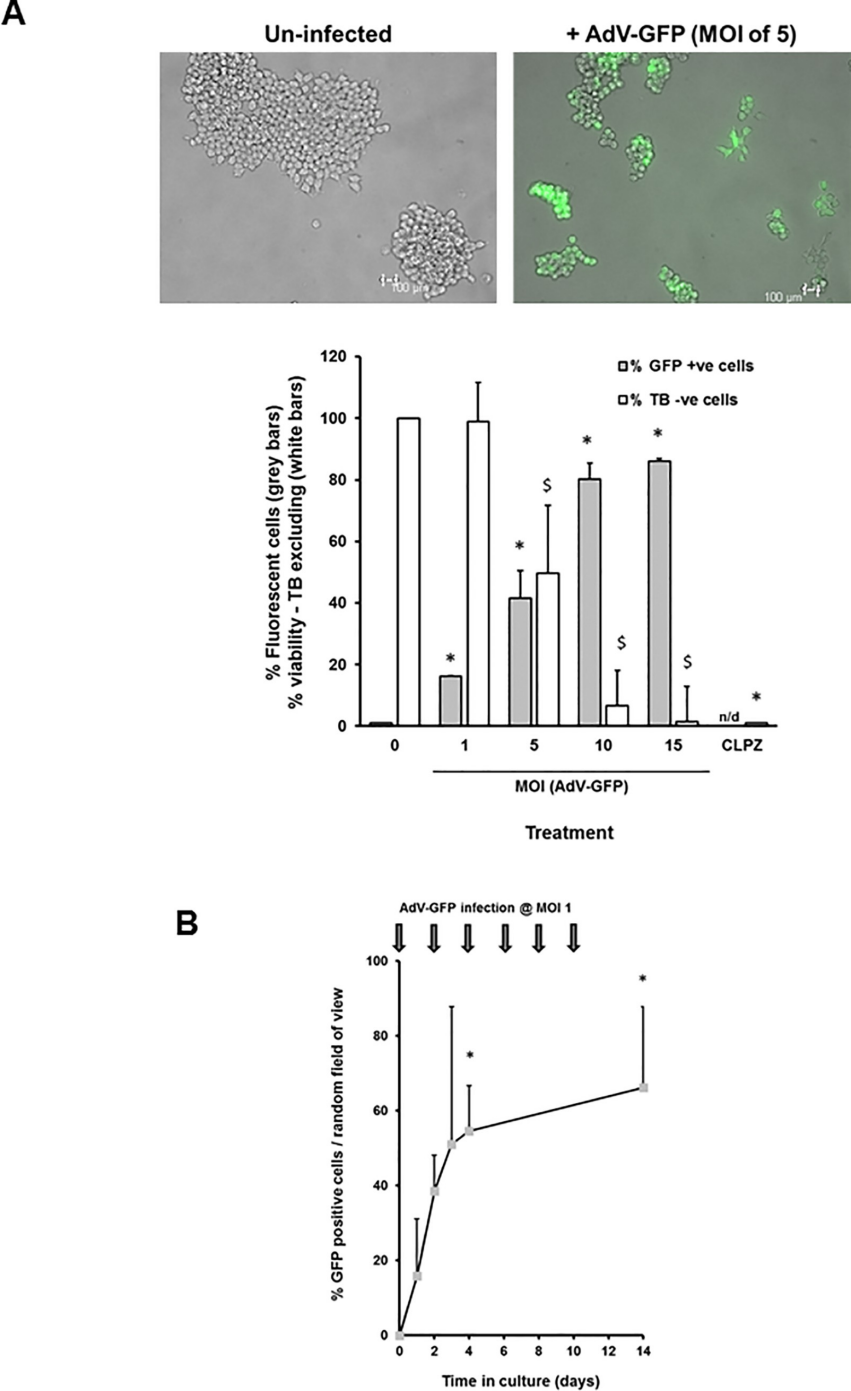
Infecting B-13 cells with AdV-GFP. **A**, Infection of B-13 cells with AdV-GFP results in expression of GFP but impacts on cell viability. B-13 cells were infected at the indicated MOI and the mean percentage and SD of cells expressing GFP determined by fluorescence microscopy from 3 randomly selected views. At day 6, the cells were incubated with trypan blue and the mean percentage and SD of cells excluding the dye determined from 3 randomly selected views. Upper panels, typical view of cells after 24 hours after infection as indicated. Lower panel, percentage cells GFP positive (grey bars) and percentage viable cells. n/d, not determined; CLPZ, 200μM chlorpromazine treatment; TB, 0.2% (w/v) trypan blue. Data typical of 3 separate experiments. *Significantly different infection from uninfected cells using one way ANOVA. ^$^Significantly different trypan blue exclusion (using one way ANOVA. **B**, Effect of repeated infection of B-13 cells with AdV-GFP on the expression of GFP. B-13 cells were infected at an MOI of 1.0 as indicated by the arrows and the mean percentage and SD of cells expressing GFP determined by fluorescence microscopy from 3 randomly selected views. Data typical of 3 separate experiments. *Significantly different from time zero uninfected cells using one way ANOVA.

To increase the likelihood that a significant proportion of cells express SGK1F, an adenoviral vector system was employed. Fig 1A demonstrates that in excess of 80% of B-13 cells were readily infected with an adenovirus encoding green fluorescent protein (AdV-GFP) at an MOI of 15, however infection levels greater than an MOI of 1 also lead to a dose-dependent increase in cell death by day 6. Since infection at an MOI of 1 resulted in moderate (~20%) infection with minimal toxicity, repeated infection at an MOI of 1 was employed to maximize the proportion of cells infected whilst minimizing toxicity (Fig 1B). This approach was successful for around 10 days but typically, significant toxicity after this time was observed, which limited scope for testing effects of SGK1F expression.

**Fig 2:**
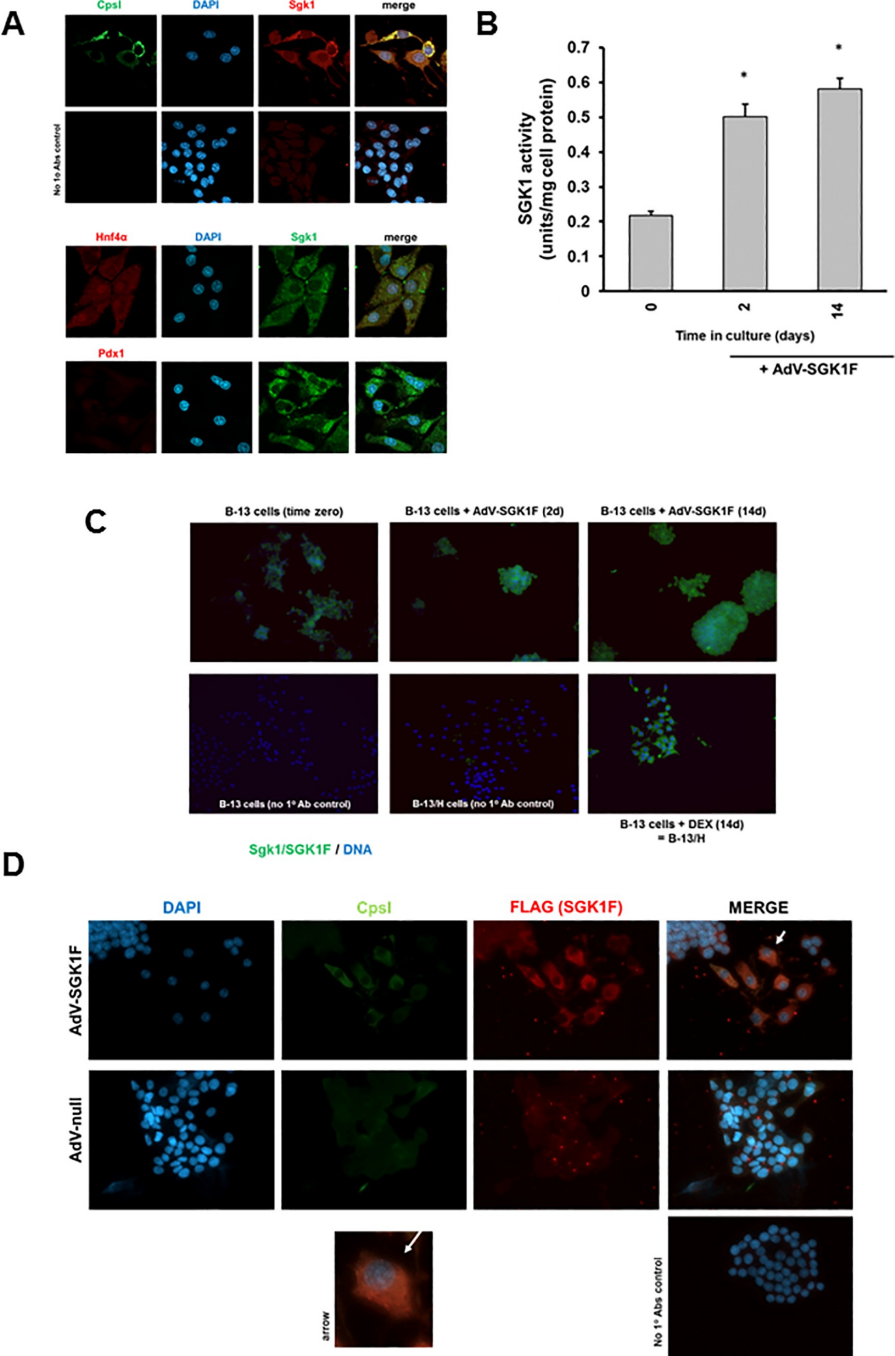
Expression of B-13-derived Sgk1 and AdV-SGK1F in B-13 cells. **A,** confocal images of B-13/H cells produced through exposure to10nM dexamethasone glucocorticoid and examined for the expression of CpsI and Sgk1 (upper panels) or co-expression of either Hnf4α or Pdx1 with Sgk1 (lower panels). Data are typical of 4 separate experiments. All images were taken and under identical spectral conditions and processed identically. **B**, Infection with AdV-SGK1F results in an increase in SGK1 kinase activity. B-13 cells were repeatedly infected with AdV-SGK1F using the repeated infection at an MOI of 1 protocol and SGK1 kinase activity determined in cell extracts as outlined in the methods section. Data are the mean and SD of 3 replicates from the same experiment, typical of 3 separate experiments. **C**, Immunocytochemical detection of SGK1 expression. B-13 cells were repeatedly infected with AdV-SGK1F at an MOI of 1, cells fixed and Sgk1/SGK1F expression (green) determined by immunocytochemistry with DAPI (blue) for identification of nuclei. Data typical of 3 separate experiments. All images were taken and under identical spectral conditions and processed identically. **D**, Immunocytochemical detection of CpsI and SGK1-F expression. B-13 cells were repeatedly infected with AdV-SGK1F using repeated infection at an MOI of 1, cells fixed and CpsI (green) and SGK1F tag expression (red) determined by immunocytochemistry with DAPI (blue) for identification of nuclei. Note, the cells were not treated with DEX. Data typical of 3 separate experiments. All images were taken and under identical spectral conditions and processed identically. Panel labelled “arrow” is an expanded view of the cell identified by an arrow in the top right hand panel.

Carbamoyl phosphate synthase (CpsI) is an hepatocyte-specific gene [28] and Fig 2A demonstrates—using the standard glucocorticoid-dependent differentiation protocol (14 days treatment with 10nM dexamethasone (DEX))—expression of both CpsI and Sgk1 in B-13/H cells, with higher punctate staining (likely due mitochondrial localization) correlating with cells expressing the highest levels of Sgk1 protein. B-13/H cells also expressed Hnf4α, most highly in the nuclei of cells, whereas the pancreatic beta cell marker Pdx1, was expressed at relatively low (with a nuclear presence) to undetectable levels (Fig 2A).

Employing the repeated infection strategy in B-13 cells (in the absence of DEX) using AdV-SGK1F resulted detectable increases in Sgk1/SGK1F kinase activity (Fig 2B); widespread increased expression of Sgk1/SGK1F in infected remaining viable cells (Fig 2C) and co-expression of CpsI with SGK1F-expressing cells (determined specifically using the N terminal tag sequence cloned into the protein) (Fig 2D).

**Fig 3:**
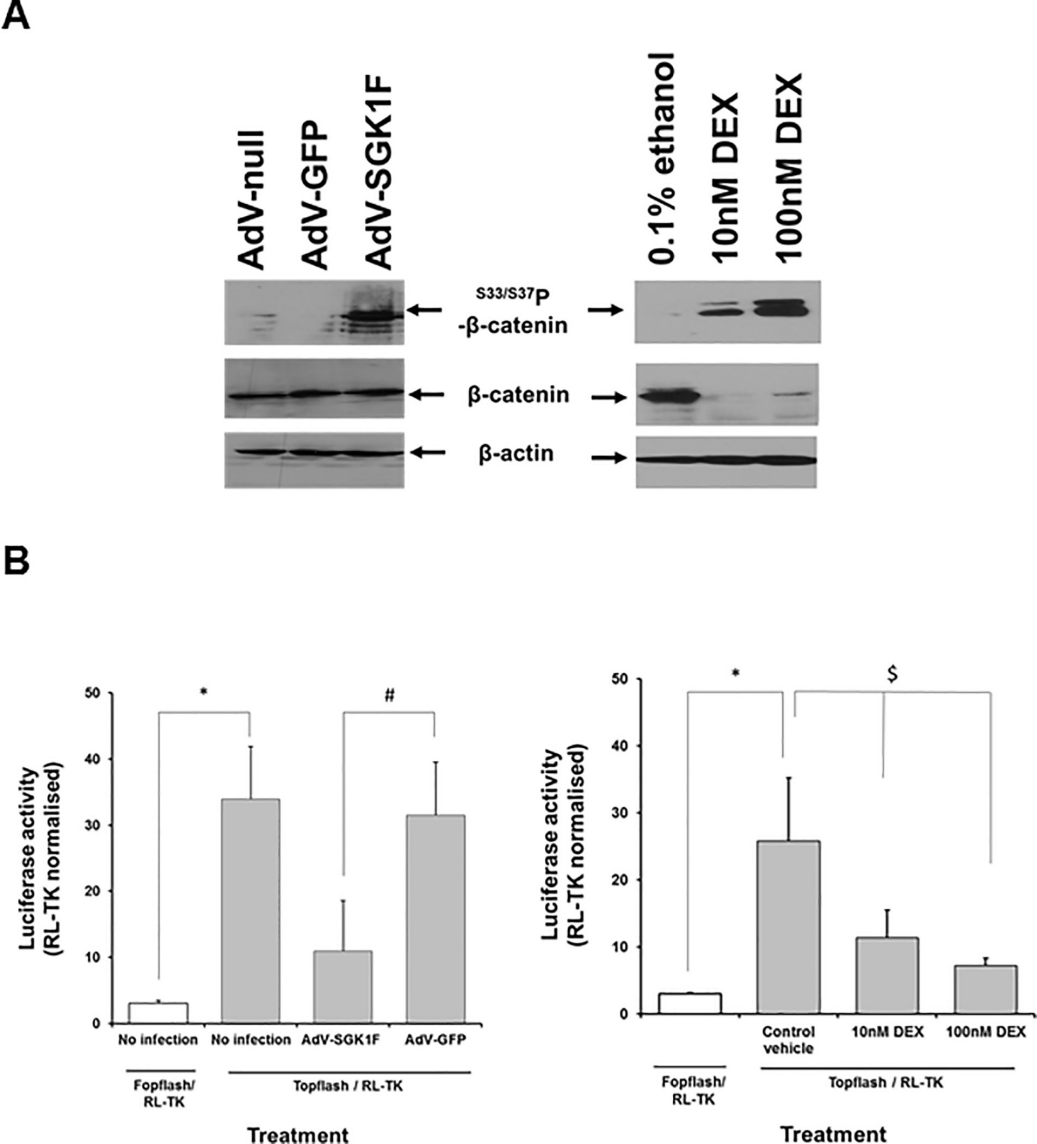
AdV-SGK1F expression in B-13 cells results in a repression in WNT signalling activity. **A**, Phosphorylation of β-catenin in B-13 cells in response to AdV-SGK1F infection. B-13 cells were repeatedly infected with AdV-SGK1F or the control AdVs (AdV-GFP, AdV-null) at an MOI of 5 over a 4 day period (left panel) or treated with the indicated concentration of DEX for 14 days (right panel) before expression levels of the indicated proteins were determined by Western blot (20μg cell protein/lane). Data typical of 3 separate experiments. **B**, Infecting B-13 cells with AdV-SGK1F leads to reduction in Tcf/Lef (Wnt signalling) transcriptional activity. Topflash is a construct containing two sets (with the second set in the reverse orientation) of three copies of the TCF-binding site upstream of a thymidine kinase promoter and luciferase open reading frame. Fopflash is an identical vector to Topflash, but with mutated TCF response elements [14]. Twenty four hours after transfection, cells were either infected with the indicated AdV at an MOI of 5 (left panel only) or treated with DEX or vehicle control as indicated (right panel). After a further 24 hours, cells were lysed and the levels of luciferase and renilla determined as outlined in the methods section. Data are the mean and SD of 5 separate determinations from the same experiment, typical of 3 separate experiments. Significantly different versus *Fopflash or ^#^AdV-GFP infected cells using the Student’s T test (two tailed).

Infection of B-13 cells also resulted in changes in the ratio of β-catenin/phosphorylated-β-catenin (Fig 3A) and suppression of WNT signaling activity (Fig 3B), as observed with glucocorticoid exposure [14].

**Fig 4:**
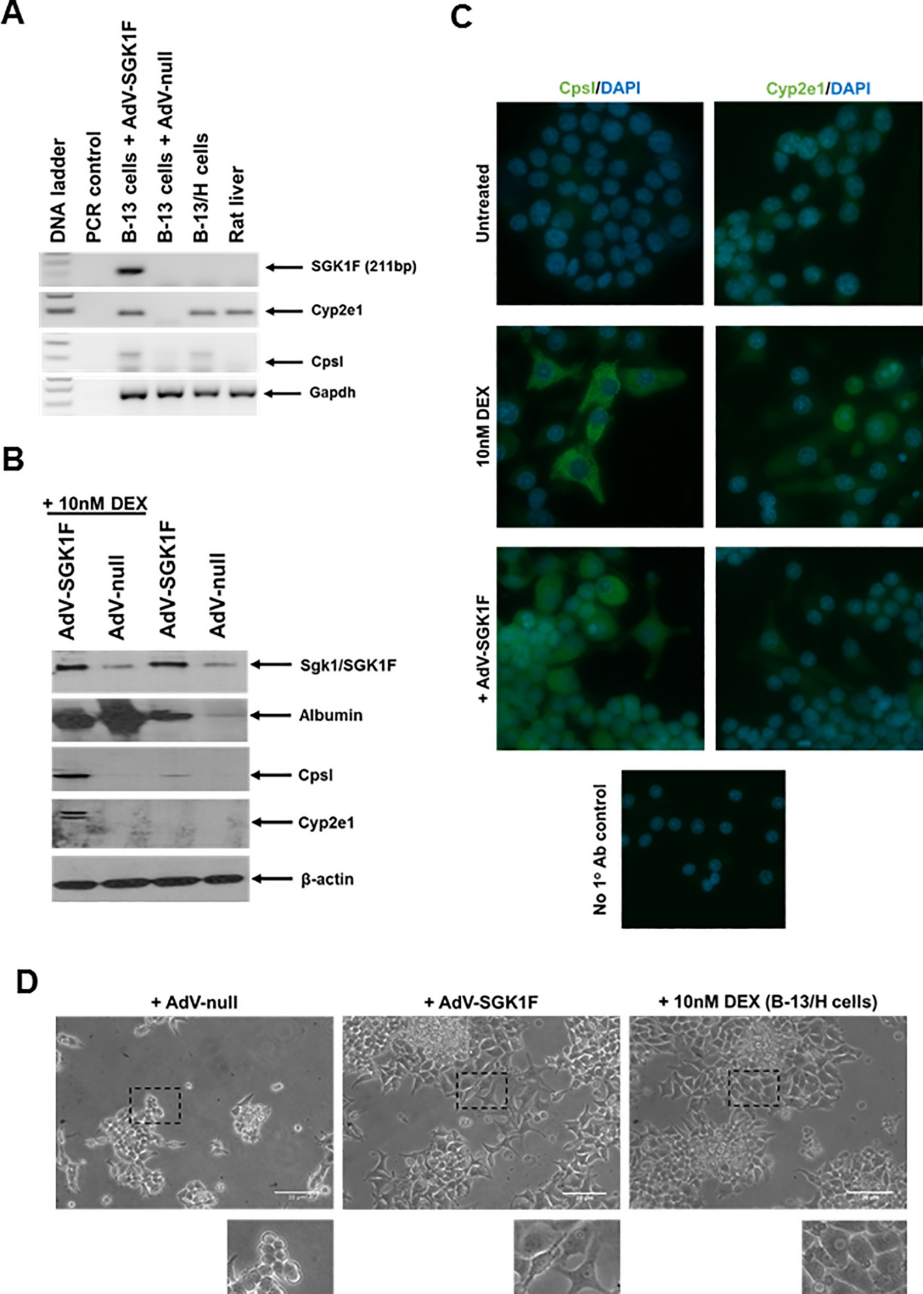
Infecting B-13 cells with AdV-SGK1F leads to the expression of hepatic markers and a morphological change into the B-13/H phenotype. **A**, Transcript expression. B-13 cells were repeatedly infected with the indicated AdV before total RNA was isolated at day 8 and transcript expression determined by RT-PCR as outlined in methods section. Data are typical of 3 separate experiments. **B**, Protein expression. B-13 cells were repeatedly infected with the indicated AdV (with or without co-treatment with DEX as indicated) before indicated protein expression was determined by Western blotting at day 8. Data are typical of 3 separate experiments. **C**, Immunocytochemical detection of CpsI and Cyp2e1 expression. B-13 cells were repeatedly infected with AdV-SGK1F (MOI of 5) or treated with 10nM DEX for 14 days (to generate B-13/H cells) and cells fixed and indicated protein expression (green) determined by immunocytochemistry with DAPI (blue) for identification of nuclei. Data typical of 3 separate experiments. All images were taken and under identical spectral conditions and processed identically. **D**, Morphology of B-13 cells after infection with AdV-SGK1F. Light micrographs of B-13 cells infected with the indicated AdV (MOI of 5) or treated with 10nM DEX for 14 days (B-13/H cells). Untreated and ethanol vehicle treated appear identically to AdV-null treated cells and are not shown for brevity.

The adoption of an hepatocyte-like phenotype by B-13 cells in response to AdV-SGK1F infection was confirmed by detection of liver-enriched and liver-specific gene transcripts Cyp2e1 and CpsI respectively, similarly to cells treated with dexamethasone for 14 days (Fig 4A). Fig 4B demonstrates that the effects of SGK1F expression also extended to detectable levels of hepatocyte marker proteins (with the exception of Cyp2e1), detectable expression of CpsI was observed in the majority of the cells (Fig 4C) and a morphological change from B-13 cells (Fig 4D). However, although some morphological changes are shared with B-13 cells treated with DEX such as enlargement and visible nucleus, there were also clear differences (Fig 4D), suggesting that DEX treatment may have effects on B-13 cells in addition to those of Sgk1 induction critical for full conversion to hepatocyte-like (B-13/H) cells.

These data support our proposal that glucocorticoid-dependent induction of Sgk1 in B-13 cells is a critical downstream event in B-13 cell conversion to B-13/H cells and likely involves cross-talk with the WNT signaling pathway [15], since expression of SGK1F in B-13 cells resulted in conversion to a phenotype near-similar to B-13/H cells in the absence of glucocorticoid,

### Early expression of SGK1F in iPSCs directed to differentiate into endoderm has no effect on progression to endodermal cells

Since expression of SGK1F in B-13 cells resulted in conversion of B-13 cells to B-13/H cells, these data also confirm functionality of the adenoviral-derived SGK1F protein. The effect of SGK1F expression in iPSCs directed to differentiate into hepatocytes was therefore examined.

**Fig 5:**
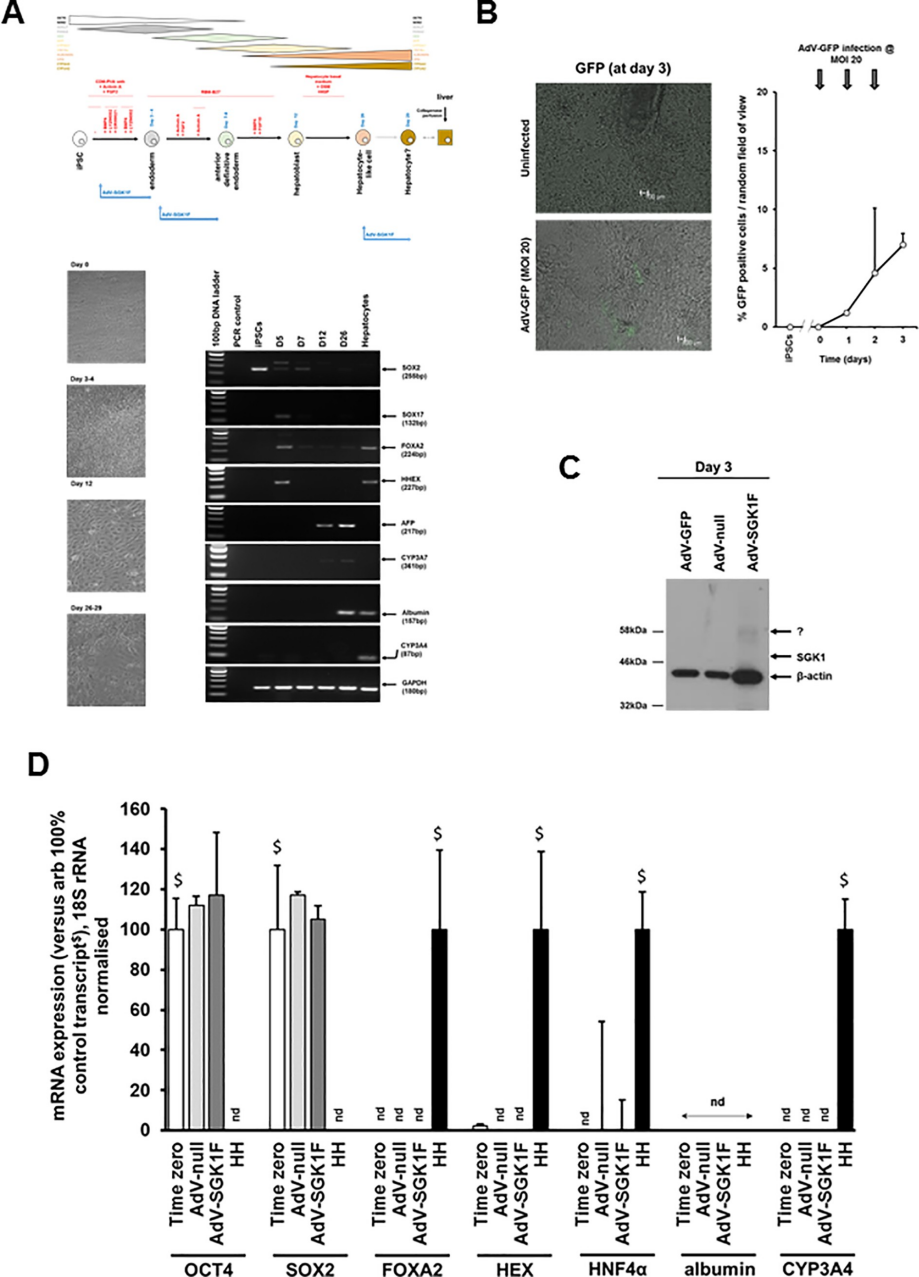
Early expression of SGK1F in iPSCs directed to differentiate into endoderm has no effect on progression to endodermal cells. **A**, Upper panel, schematic diagram depicting the protocol [25] employed to direct iPSCs toward hepatocyte-like cells. Lower left panel, light micrographs of iPSCs at different stages–as indicated—of differentiation to hepatocyte-like cells. Lower right panel, RT-PCR for selected transcripts in iPSCs at the different stages of differentiation to hepatocyte-like cells. Results typical of 10 separate experiments. Note, the cells were not infected with AdV-SGK1F. **B**, Effect of repeated infection of iPSCs after 1 day of differentiation with AdV-GFP on the expression of GFP. iPSCs were infected at an MOI of 20.0 and the mean percentage and SD of cells expressing GFP determined by fluorescence microscopy from 3 randomly selected views up to 3 days later. Typical views shown. Data typical of 3 separate experiments. **C**, Western blot analysed for the expression of flag tagged SGK1F, followed by β-actin. Cells were repeatedly infected at day 0 at an MOI of 20.0 with the indicated AdV construct prior to harvest at day 3 and total cell protein analysis by Western blotting. Each lane contained 20 μg protein/lane. **D**, Transcript expression. iPSCs after 1 day of differentiation were infected with the indicated AdV before total RNA was isolated at day 3 and transcript expression determined by qRT-PCR as outlined in methods section. Data are typical of 3 separate experiments.

Employing the hepatocyte differentiation protocol outlined in the methods section and schematically described in Fig 5A, Fig 5B demonstrates that infection of differentiating iPSCs (from day 1) at an MOI of 20 gave rise to low infection rates typically in the mean range of 7% by day 3.

**Fig 6:**
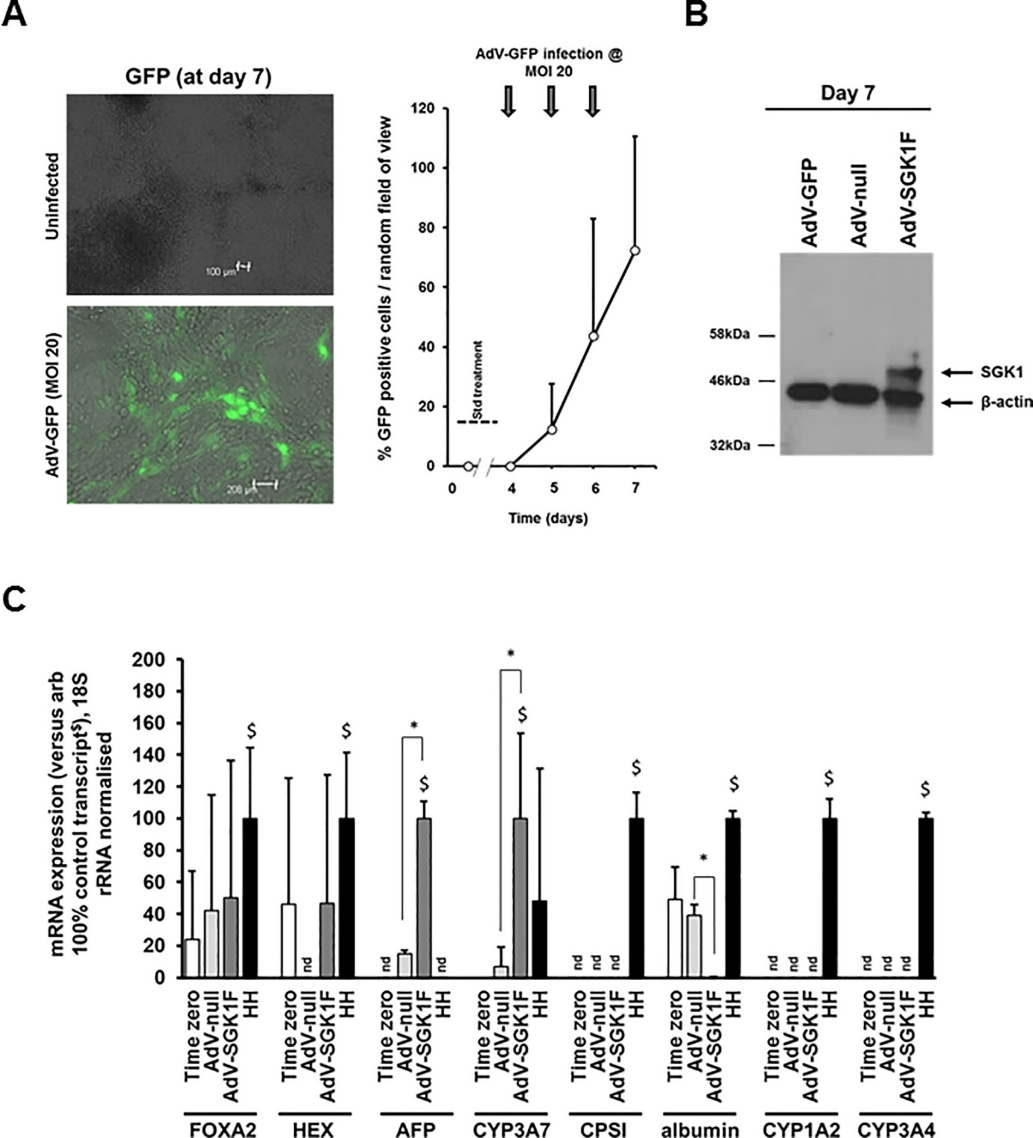
Expression of SGK1F in iPSCs-derived endoderm directed to differentiate into hepatoblasts promotes an hepatoblast phenotype. **A**, Effect of repeated infection of iPSCs after 5 days of differentiation with AdV-GFP on the percentage of cells expressing GFP at the indicated timepoints. iPSCs were infected at an MOI of 20 and the mean percentage and SD of cells expressing GFP determined by fluorescence microscopy from 3 randomly selected views. Typical view shown. Data typical of 3 separate experiments. **B**, Western blot analysed for the expression of flag tagged SGK1F, followed by β-actin. Cells were repeatedly infected at day 4 at an MOI of 20.0 with the indicated AdV construct prior to harvest at day 7 and total cell protein analysis by Western blotting. Each lane contained 20 μg protein/lane. **C**, Transcript expression. iPSCs after 5 days of differentiation were infected with the indicated AdV before total RNA was isolated at day 7 and transcript expression determined by qRT-PCR as outlined in methods section. Data are typical of 3 separate experiments.

**Fig 7:**
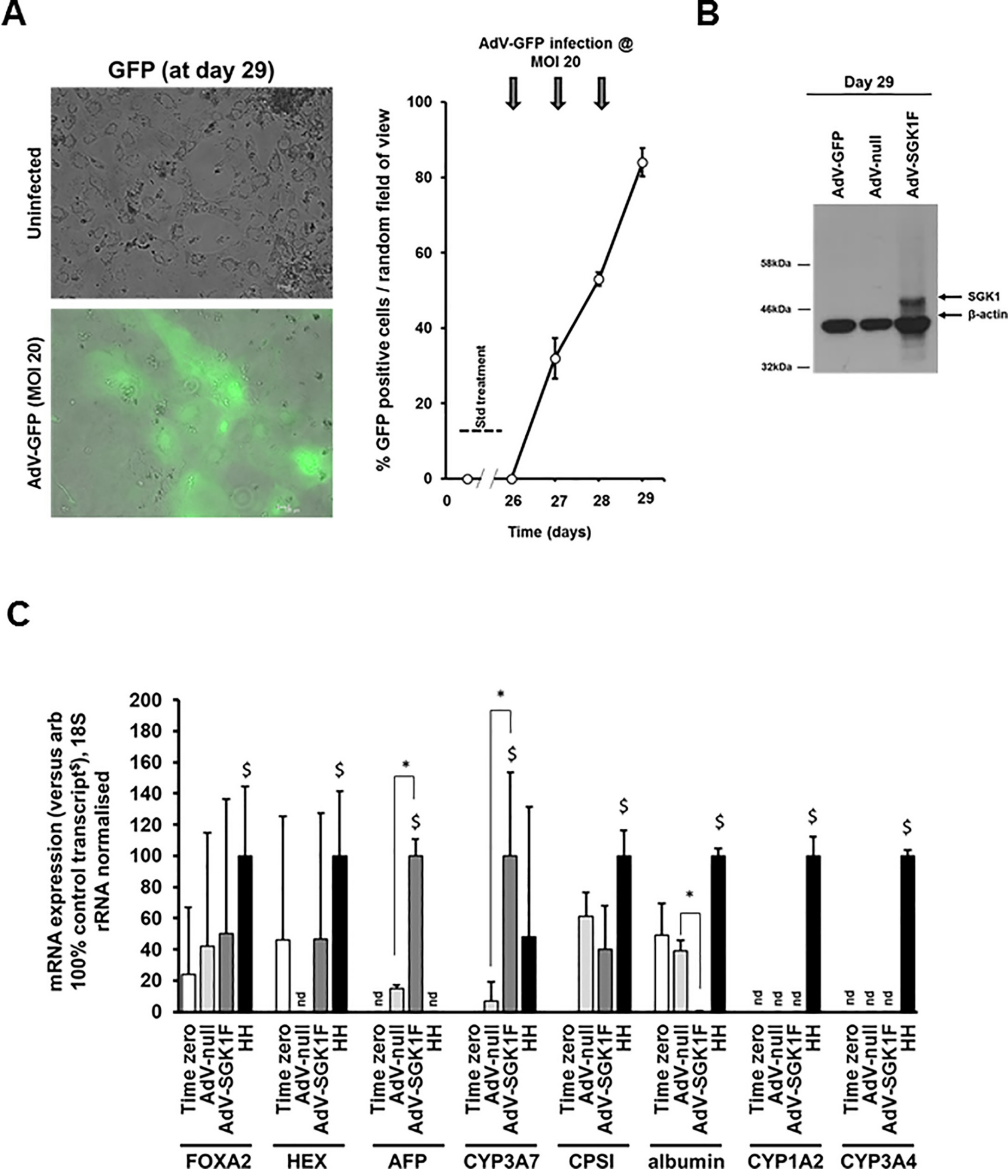
Expression of SGK1F in iPSC-derived hepatocytes results in promotion back to an hepatoblast phenotype. **A**, Effect of repeated infection of iPSCs after 26 days of differentiation with AdV-GFP on the percentage of cells expressing GFP at the indicated timepoints. iPSC-derived hepatocytes were infected at an MOI of 20 and the mean percentage and SD of cells expressing GFP determined by fluorescence microscopy from 3 randomly selected views. Typical views shown. Data typical of 3 separate experiments. **B**, Western blot analysed for the expression of flag tagged SGK1F, followed by β-actin. Cells were repeatedly infected at day 26 at an MOI of 20.0 with the indicated AdV construct prior to harvest at day 29 and total cell protein analysis by Western blotting. Each lane contained 20 μg protein/lane. **C**, Transcript expression. iPSC-derived hepatocytes after 26 days of differentiation were infected with the indicated AdV before total RNA was isolated at day 32 and transcript expression determined by qRT-PCR as outlined in methods section. Data are typical of 3 separate experiments.

Infecting cells with AdV-SGK1F at day 1 when the cells are at a pluripotent/early transition to an endoderm stage of development resulted in low levels of expression of a protein immunoreactive to the mouse anti-SGK1 antibody (Fig 5C). However, the size of this protein (~ 57 kDa) differed to the predicted size of the tagged SGK1F protein (48.9 kDa), as observed in cells directed further along the pathway to hepatocyte cells or adult hepatocytes (See Figs 6B and 7B). Using qRT-PCR to quantify pluripotency (OCT4, SOX2), endodermal (SOX17), anterior definitive endoderm (HHEX), hepatoblast (HNF4α, AFP) and mature hepatocyte (CYP3A4) at day 3, Fig 5D indicates SGK1F expression resulted in no significant changes in any transcript level (when compared to AdV-null control).

These data suggest that early expression of SGK1F in iPSCs directed to differentiate into endoderm has no effect on their differentiation to this differentiation state, either because of low rates of infection (resulting in low levels of expression) and/or because any expressed protein is rapidly degraded.

### Expression of SGK1F in iPSCs-derived endoderm directed to differentiate into anterior definitive endoderm and hepatoblasts results in promotion to an hepatoblast phenotype

Infecting cells with AdV-GFP at day 4–5, when the cells are transiting between endodermal/anterior definitive endoderm stages of development at an MOI of 20 was able to give rise to infection rates typically in the mean range of approximately 70% by day 7 based on GFP expression (Fig 6A). Infection with AdV-SGK1F during this period gave rise to a robust expression of a protein in the range of the predicted 48.9 kDa size for the tagged SGK1F protein (Fig 6B). Using qRT-PCR to quantify hepatoblast (AFP, CYP3A7) and mature hepatocyte (CPSI, albumin, CYP1A2 and CYP3A4) at day 7, Fig 6C indicates SGK1F expression significantly increased hepatoblast transcript levels but had no effect on mature transcript levels (when compared to AdV-null control).

### Late expression of SGK1F in iPSC-derived hepatocytes results in promotion back to an hepatoblast phenotype

Infecting cells with AdV-GFP at day 26, when the cells are at a post-hepatoblast / foetal hepatocyte (hepatocyte-like) stage of development—at an MOI of 20—was able to give rise to infection rates typically in the mean range of approximately 80% by day 29 based on GFP expression (Fig 7A) and robust expression of the 48.9 kDa tagged SGK1F protein (Fig 7B). Using qRT-PCR to quantify hepatoblast (AFP, CYP3A7) and mature hepatocyte (CPSI, albumin, CYP1A2 and CYP3A4) at day 29, Fig 7C indicates SGK1F expression significantly increased the hepatoblast transcripts AFP and CYP3A7. In addition, there was a significant decrease in the mature transcript albumin, and a tendency–though not significantly–for a reduction in CPSI transcripts. In contrast, the adult mature hepatocyte transcripts were not reliably detectable in iPSC-derived hepatocyte-like cells (Fig 7C). Notably, SGK1F expression did not promote a more mature phenotype in hepatoblasts directed to differentiate towards a mature hepatocyte phenotype.

These data suggest that expression of SGK1F in iPSCs differentiating toward hepatocyte-like cells promotes a reversal to an hepatoblast phenotype.

## Discussion

**Fig 8:**
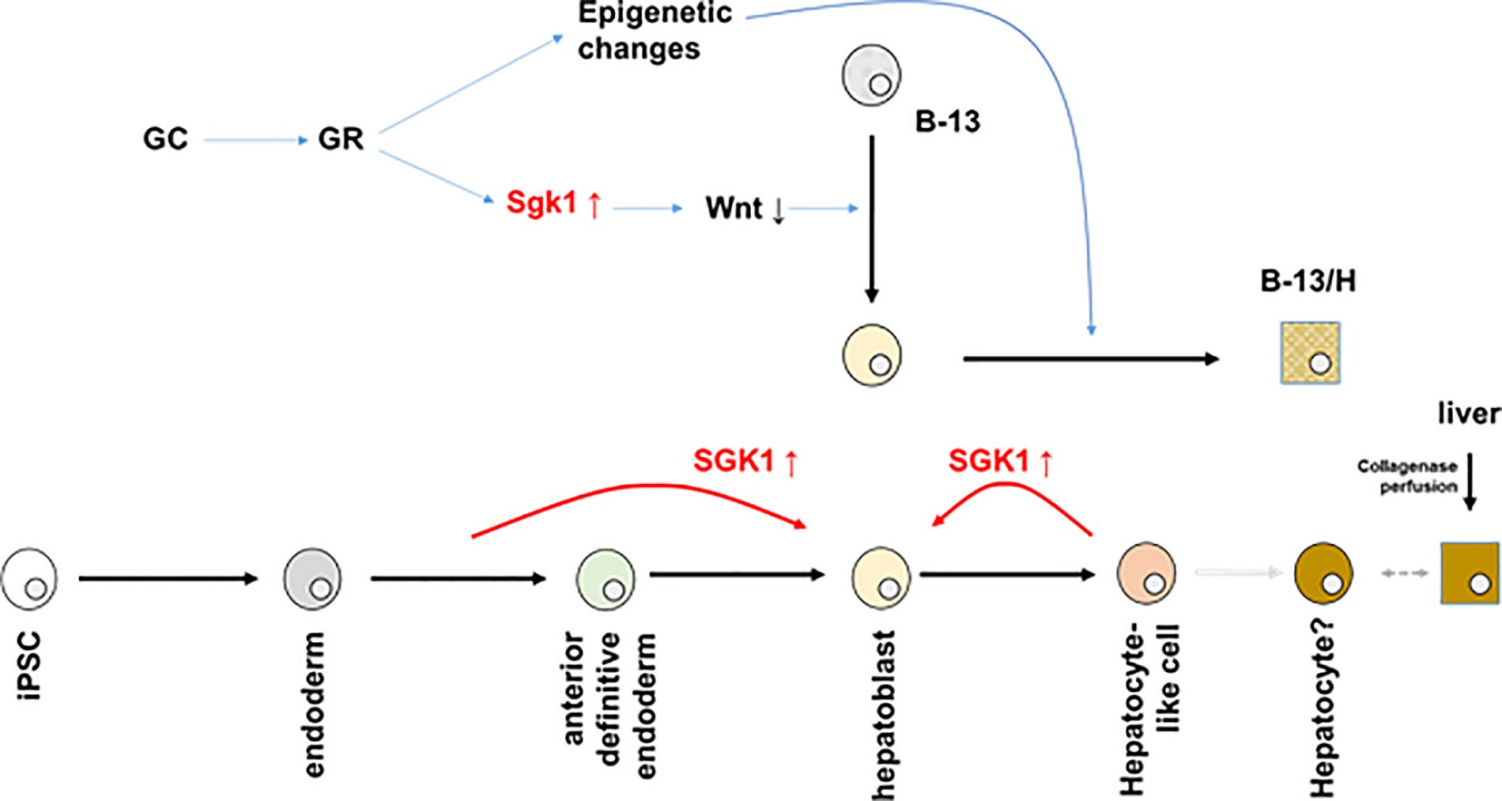
Proposed role of Sgk1 in B-13 differentiation and effects of SGK1F on human iPSC differentiation.

The rat Sgk1 gene is currently known to encode 3 validated mRNA transcripts and likely 1 further transcript [NCBI database, see also Table 2]. All 4 transcripts encode an identical core amino acid sequence and differ only in the their N terminal amino acid sequences. It is not known whether the Sgk1 isoforms have different functions, however, only the rat Sgk1c transcript appears to be irreversibly induced in B-13 cells after exposure to glucocorticoid [16]. This suggests that endogenous Sgk1c may be responsible for its differentiating effects in B-13 cells. This is supported by the high expression of the murine orthologue in pancreatic tissue from mice with high circulating endogenous glucocorticoids (these mice experience a conversion of the pancreatic exocrine tissue into hepatocyte-like cells [29]). Since expression of plasmid-encoded human-specific SGK1F protein promoted B-13 cell differentiation to B-13/H cells [15], the cDNA for this transcript was cloned into a replication-deficient adenoviral genome. The rationale for focusing on SGK1F was also driven by the knowledge that the pancreas from a patient treated for many decades with systemic glucocorticoid experienced a degree of hepatic differentiation and also contained high levels of SGK1F mRNA transcripts [27]. The data in this paper demonstrate that adenoviral-mediated SGK1F expression in B-13 cells induces their differentiation into B-13/H cells, similarly to their response to exposure to glucocorticoid. These data therefore support our proposed glucocorticoid-dependent mechanism for converting B-13 cells into B-13/H cells, as schematically outlined in Fig 8, involving glucocorticoid receptor activation, Sgk1 induction, phosphorylation of β-catenin and a suppression of endogenously high Wnt signaling activity in B-13 cells.

**Table 2:** Rat and human Sgk1 proteins.

Rat				Human			
Transcript	NCBI #	N terminal amino acid sequence encoded (total amino acids in protein; theoretical molecular weight)	Trivial name	Transcript	NCBI #	N terminal amino acid sequence encoded (total amino acids in protein; theoretical molecular weight)	Trivial name
Sgk1 isoform 1	NM_001193568	MGEMQGALARARLESLLRPRHKKRVEAQKRSESVLLSGL (445; 50627 Da)	Sgk1b	SGK1 isoform 4	NM_001143678	MGEMQGALARARLESLLRPRHKKRAEAQKRSESFLLSGL (445; 50623 Da)	SGK1B
Sgk1 isoform 2	NM_001193569	MREEALRSPWK (417; 47626 Da)	Sgk1c				
Sgk1 isoform 3	NM_019232	MTVKTEAARSTLTYSRMRGMVAILI (431; 49024 Da)	Sgk1a	SGK1 isoform 1	NM_005627	MTVKTEAAKGTLTYSRMRGMVAILI (431; 48942 Da)	SGK1A
				SGK1 isoform 5	NM_001291995	MTVKTEAAKGTLTYSRMRGMVAILI (387; 43811 Da)	
Sgk1 isoform X1	XM_006227723	MVNKDMNGFPVKKCSAFQFFKKRVRRWIKSPMVSVDKHQSPNLKYTGPAGVHLPPGEPDFEPALCQSCLGDHTFQRGMLSPEESRSWEIQPGGEVKEPCNHANILTKPDPRTFWTSDDP (525; 59721 Da)		SGK1 isoform 2	NM_001143676	MVNKDMNGFPVKKCSAFQFFKKRVRRWIKSPMVSVDKHQSPSLKYTGSSMVHIPPGEPDFESSLCQTCLGEHAFQRGVLPQENESCSWETQSGCEVREPCNHANILTKPDPRTFWTNDDP (526; 59903 Da)	SGK1D
				SGK1 isoform 3	NM_001143677	MSSQSSSLSEACSREAYSSHNWALPPASRSNPQPAYPWATRRMKEEAIKPPLK (459; 52119 Da)	SGK1C
				-	GenBank: CAR58098.1	MKPSKRFFISPPSST (421; 47910 Da)	SGK1F
				-	-	MDYKDDDDKKPSKRFFISPPSST (429; 48905 Da)	AdV-encoded SGK1F

Underlined sequence, flag sequence. For raw sequence data, see below. For further details, see S1 Fig.

The effect of expressing SGK1F in human iPSC-derived cells contrasts with its effects when expressed in B-13 cells. Although a time course and dose-dependence (i.e. through assessing effects of MOI on maturation) for the expression in iPSCs was not undertaken due to limitations in stem cells, it is likely SGK1F was readily and rapidly expressed in some cells since, for example, robust expression was seen 3 days after infection (Fig 7C). Indeed, reading across to GFP expression–which can be monitored in a non-destructive fashion—expression via AdV-GFP occurs within 24 hours in all iPSC-derived cells since GFP is detectable in infected cells within 24 hours (Fig 5B, Fig 6A and Fig 7A). However. the data in this report suggest that progression to endoderm was not affected by expression of SGK1F soon after stimulation to differentiate from stem cells into endoderm although this may be related to low levels of infection of cells at this stage of development. Therefore, it could not be reliably determined whether expression of SGK1F affected progression of iPSC differentiation into endoderm. Assessing the effects of different MOI (i.e. gene dose) would strengthen this observation, in addition to such an application at stages of maturation where iPSC-derived cells were more readily infected. However, expression of SGK1F in iPSCs-derived endoderm directed to differentiate into hepatoblasts resulted in promotion to an hepatoblast phenotype. Despite this maturing effect in endoderm, late expression of SGK1F in iPSCs-derived hepatocytes resulted in an enhanced hepatoblast phenotype. On this basis therefore, SGK1F expression does not act to promote an increase maturation of stem cell-derived terminally differentiating hepatocytes. Rather, SGK1F expression promoted the formation of hepatoblasts from both endoderm and hepatocytes. Accordingly, these effects in stem cells suggest that the role of Sgk1 in B-13 cell differentiation to B-13/H cells is likely associated with an induction of an (transient) hepatoblast-like phenotype. Previous investigations in B-13 cells suggest that Sgk1 cross-talks with the Wnt signalling pathway through phosphorylation of β-catenin and thereby to reduced Tcf/Lef transcriptional activity [14]. However, it was also noted that these effects were transient and that Wnt signalling activity (and Wnt3a expression, likely acting in an autocrine fashion in B-13 cells) returned to near B-13 levels in B-13/H cells.

Interesting, both RT-PCR (Fig 5A) and qRT-CR data (Fig 6C and Fig 7C) show relatively high levels of FOXA2 and HEX expression in human hepatocytes compared to later maturing iPSC-derived anterior definitive endoderm and hepatocyte-like cells. Hepatocytes from this lab are currently isolated from livers that–after a cold preservation period of at least 24 hours–undergo a warm re-conditioning perfusion period of up to 24 hours prior to a collagenase perfusion (for more details, see [30]). The isolation procedure alone, is known to significantly modulate gene expression in the rat [31]. The high levels of apparently mature hepatocyte FOXA2 and HEX transcripts may therefore be associated with an already advance de-differentiation process.

## Conclusion

This paper reports for the first time that SGK1F expression in iPSCs promotes an hepatoblast phenotype and by implication, that this hepatoblast promotion is likely the major role played by glucocorticoid-induced Sgk1c expression in B-13 cells. These insights could be exploited to modulate expression to i) enhance the early stages of iPSC differentiation into hepatoblasts and ii) enhance B-13 conversion into B-13/H cells to promote hepatocyte phenotype.

## Supporting information

S1 Fig Comparison of rat and human Sgk1 proteins. (DOCX)
