# Supplementary material for: Expression of serine/threonine protein kinase SGK1F promotes an hepatoblast state in stem cells directed to differentiate into hepatocytes
Source: PLoS One. 2019 Jun 26;14(6):e0218135. doi: 10.1371/journal.pone.0218135 (PMC6594595; doi:10.1371/journal.pone.0218135)
Supplement: S1 Fig — (DOCX) [file pone.0218135.s001.docx]

**S1 Fig Comparison of rat and human Sgk1 proteins**

**Rat Sgk1 protein sequences**

>NP_001180497.1 serine/threonine-protein kinase Sgk1 isoform 1 [Rattus norvegicus]

MGEMQGALARARLESLLRPRHKKRVEAQKRSESVLLSGLAFMKQRRMGLNDFIQKLANNSYACKHPEVQSYLKISQPQEPELMNANPSPPPSPSQQINLGPSSNPHAKPSDFHFLKVIGKGSFGKVLLARHKAEEAFYAVKVLQKKAILKKKEEKHIMSERNVLLKNVKHPFLVGLHFSFQTADKLYFVLDYINGGELFYHLQRERCFLEPRARFYAAEIASALGYLHSLNIVYRDLKPENILLDSQGHIVLTDFGLCKENIEHNGTTSTFCGTPEYLAPEVLHKQPYDRTVDWWCLGAVLYEMLYGLPPFYSRNTAEMYDNILNKPLQLKPNITNSARHLLEGLLQKDRTKRLGAKDDFMEIKSHIFFSLINWDDLINKKITPPFNPNVSGPSDLRHFDPEFTEEPVPSSIGRSPDSILVTASVKEAAEAFLGFSYAPPMDSFL

>NP_001180498.1 serine/threonine-protein kinase Sgk1 isoform 2 [Rattus norvegicus]

MREEALRSPWKAFMKQRRMGLNDFIQKLANNSYACKHPEVQSYLKISQPQEPELMNANPSPPPSPSQQINLGPSSNPHAKPSDFHFLKVIGKGSFGKVLLARHKAEEAFYAVKVLQKKAILKKKEEKHIMSERNVLLKNVKHPFLVGLHFSFQTADKLYFVLDYINGGELFYHLQRERCFLEPRARFYAAEIASALGYLHSLNIVYRDLKPENILLDSQGHIVLTDFGLCKENIEHNGTTSTFCGTPEYLAPEVLHKQPYDRTVDWWCLGAVLYEMLYGLPPFYSRNTAEMYDNILNKPLQLKPNITNSARHLLEGLLQKDRTKRLGAKDDFMEIKSHIFFSLINWDDLINKKITPPFNPNVSGPSDLRHFDPEFTEEPVPSSIGRSPDSILVTASVKEAAEAFLGFSYAPPMDSFL

>NP_062105.2 serine/threonine-protein kinase Sgk1 isoform 3 [Rattus norvegicus]

MTVKTEAARSTLTYSRMRGMVAILIAFMKQRRMGLNDFIQKLANNSYACKHPEVQSYLKISQPQEPELMNANPSPPPSPSQQINLGPSSNPHAKPSDFHFLKVIGKGSFGKVLLARHKAEEAFYAVKVLQKKAILKKKEEKHIMSERNVLLKNVKHPFLVGLHFSFQTADKLYFVLDYINGGELFYHLQRERCFLEPRARFYAAEIASALGYLHSLNIVYRDLKPENILLDSQGHIVLTDFGLCKENIEHNGTTSTFCGTPEYLAPEVLHKQPYDRTVDWWCLGAVLYEMLYGLPPFYSRNTAEMYDNILNKPLQLKPNITNSARHLLEGLLQKDRTKRLGAKDDFMEIKSHIFFSLINWDDLINKKITPPFNPNVSGPSDLRHFDPEFTEEPVPSSIGRSPDSILVTASVKEAAEAFLGFSYAPPMDSFL

>XP_006227785.1 PREDICTED: serine/threonine-protein kinase Sgk1 isoform X1 [Rattus norvegicus]

MVNKDMNGFPVKKCSAFQFFKKRVRRWIKSPMVSVDKHQSPNLKYTGPAGVHLPPGEPDFEPALCQSCLGDHTFQRGMLSPEESRSWEIQPGGEVKEPCNHANILTKPDPRTFWTSDDPAFMKQRRMGLNDFIQKLANNSYACKHPEVQSYLKISQPQEPELMNANPSPPPSPSQQINLGPSSNPHAKPSDFHFLKVIGKGSFGKVLLARHKAEEAFYAVKVLQKKAILKKKEEKHIMSERNVLLKNVKHPFLVGLHFSFQTADKLYFVLDYINGGELFYHLQRERCFLEPRARFYAAEIASALGYLHSLNIVYRDLKPENILLDSQGHIVLTDFGLCKENIEHNGTTSTFCGTPEYLAPEVLHKQPYDRTVDWWCLGAVLYEMLYGLPPFYSRNTAEMYDNILNKPLQLKPNITNSARHLLEGLLQKDRTKRLGAKDDFMEIKSHIFFSLINWDDLINKKITPPFNPNVSGPSDLRHFDPEFTEEPVPSSIGRSPDSILVTASVKEAAEAFLGFSYAPPMDSFL

**CLUSTAL O(1.2.4) multiple sequence alignment of rat Sgk1 protein sequences**

4 MVNKDMNGFPVKKCSAFQFFKKRVRRWIKSPMVSVDKHQSPNLKYTGPAGVHLPPGEPDF 60

3 ------------------------------------------------------------ 0

1 ------------------------------------------------------------ 0

2 ------------------------------------------------------------ 0

4 EPALCQSCLGDHTFQRGMLSPEESRSWEIQPGGEVKEPCNHANILTKPDPRTFWTSDDPA 120

3 ----------------------------M-----TVKTEAARSTLTYS-RMRGMVAILIA 26

1 --------MGEM---QGALARAR---LES-----LLRPRHKKRVEAQK-RSESVLLSGLA 40

2 -----------------------------------------------M-REEALRSPWKA 12

*

4 FMKQRRMGLNDFIQKLANNSYACKHPEVQSYLKISQPQEPELMNANPSPPPSPSQQINLG 180

3 FMKQRRMGLNDFIQKLANNSYACKHPEVQSYLKISQPQEPELMNANPSPPPSPSQQINLG 86

1 FMKQRRMGLNDFIQKLANNSYACKHPEVQSYLKISQPQEPELMNANPSPPPSPSQQINLG 100

2 FMKQRRMGLNDFIQKLANNSYACKHPEVQSYLKISQPQEPELMNANPSPPPSPSQQINLG 72

************************************************************

4 PSSNPHAKPSDFHFLKVIGKGSFGKVLLARHKAEEAFYAVKVLQKKAILKKKEEKHIMSE 240

3 PSSNPHAKPSDFHFLKVIGKGSFGKVLLARHKAEEAFYAVKVLQKKAILKKKEEKHIMSE 146

1 PSSNPHAKPSDFHFLKVIGKGSFGKVLLARHKAEEAFYAVKVLQKKAILKKKEEKHIMSE 160

2 PSSNPHAKPSDFHFLKVIGKGSFGKVLLARHKAEEAFYAVKVLQKKAILKKKEEKHIMSE 132

************************************************************

4 RNVLLKNVKHPFLVGLHFSFQTADKLYFVLDYINGGELFYHLQRERCFLEPRARFYAAEI 300

3 RNVLLKNVKHPFLVGLHFSFQTADKLYFVLDYINGGELFYHLQRERCFLEPRARFYAAEI 206

1 RNVLLKNVKHPFLVGLHFSFQTADKLYFVLDYINGGELFYHLQRERCFLEPRARFYAAEI 220

2 RNVLLKNVKHPFLVGLHFSFQTADKLYFVLDYINGGELFYHLQRERCFLEPRARFYAAEI 192

************************************************************

4 ASALGYLHSLNIVYRDLKPENILLDSQGHIVLTDFGLCKENIEHNGTTSTFCGTPEYLAP 360

3 ASALGYLHSLNIVYRDLKPENILLDSQGHIVLTDFGLCKENIEHNGTTSTFCGTPEYLAP 266

1 ASALGYLHSLNIVYRDLKPENILLDSQGHIVLTDFGLCKENIEHNGTTSTFCGTPEYLAP 280

2 ASALGYLHSLNIVYRDLKPENILLDSQGHIVLTDFGLCKENIEHNGTTSTFCGTPEYLAP 252

************************************************************

4 EVLHKQPYDRTVDWWCLGAVLYEMLYGLPPFYSRNTAEMYDNILNKPLQLKPNITNSARH 420

3 EVLHKQPYDRTVDWWCLGAVLYEMLYGLPPFYSRNTAEMYDNILNKPLQLKPNITNSARH 326

1 EVLHKQPYDRTVDWWCLGAVLYEMLYGLPPFYSRNTAEMYDNILNKPLQLKPNITNSARH 340

2 EVLHKQPYDRTVDWWCLGAVLYEMLYGLPPFYSRNTAEMYDNILNKPLQLKPNITNSARH 312

************************************************************

4 LLEGLLQKDRTKRLGAKDDFMEIKSHIFFSLINWDDLINKKITPPFNPNVSGPSDLRHFD 480

3 LLEGLLQKDRTKRLGAKDDFMEIKSHIFFSLINWDDLINKKITPPFNPNVSGPSDLRHFD 386

1 LLEGLLQKDRTKRLGAKDDFMEIKSHIFFSLINWDDLINKKITPPFNPNVSGPSDLRHFD 400

2 LLEGLLQKDRTKRLGAKDDFMEIKSHIFFSLINWDDLINKKITPPFNPNVSGPSDLRHFD 372

************************************************************

4 PEFTEEPVPSSIGRSPDSILVTASVKEAAEAFLGFSYAPPMDSFL 525

3 PEFTEEPVPSSIGRSPDSILVTASVKEAAEAFLGFSYAPPMDSFL 431

1 PEFTEEPVPSSIGRSPDSILVTASVKEAAEAFLGFSYAPPMDSFL 445

2 PEFTEEPVPSSIGRSPDSILVTASVKEAAEAFLGFSYAPPMDSFL 417

*********************************************

**Human SGK1 protein sequences**

Note: Transcripts from isoform 1 and isoform 5 generate the same N terminus but differ in that isoform 5 lacks an internal 44 residues present in all other proteins.

>NP_005618.2 serine/threonine-protein kinase Sgk1 isoform 1 [Homo sapiens]

MTVKTEAAKGTLTYSRMRGMVAILIAFMKQRRMGLNDFIQKIANNSYACKHPEVQSILKISQPQEPELMNANPSPPPSPSQQINLGPSSNPHAKPSDFHFLKVIGKGSFGKVLLARHKAEEVFYAVKVLQKKAILKKKEEKHIMSERNVLLKNVKHPFLVGLHFSFQTADKLYFVLDYINGGELFYHLQRERCFLEPRARFYAAEIASALGYLHSLNIVYRDLKPENILLDSQGHIVLTDFGLCKENIEHNSTTSTFCGTPEYLAPEVLHKQPYDRTVDWWCLGAVLYEMLYGLPPFYSRNTAEMYDNILNKPLQLKPNITNSARHLLEGLLQKDRTKRLGAKDDFMEIKSHVFFSLINWDDLINKKITPPFNPNVSGPNDLRHFDPEFTEEPVPNSIGKSPDSVLVTASVKEAAEAFLGFSYAPPTDSFL

>NP_001137148.1 serine/threonine-protein kinase Sgk1 isoform 2 [Homo sapiens]

MVNKDMNGFPVKKCSAFQFFKKRVRRWIKSPMVSVDKHQSPSLKYTGSSMVHIPPGEPDFESSLCQTCLGEHAFQRGVLPQENESCSWETQSGCEVREPCNHANILTKPDPRTFWTNDDPAFMKQRRMGLNDFIQKIANNSYACKHPEVQSILKISQPQEPELMNANPSPPPSPSQQINLGPSSNPHAKPSDFHFLKVIGKGSFGKVLLARHKAEEVFYAVKVLQKKAILKKKEEKHIMSERNVLLKNVKHPFLVGLHFSFQTADKLYFVLDYINGGELFYHLQRERCFLEPRARFYAAEIASALGYLHSLNIVYRDLKPENILLDSQGHIVLTDFGLCKENIEHNSTTSTFCGTPEYLAPEVLHKQPYDRTVDWWCLGAVLYEMLYGLPPFYSRNTAEMYDNILNKPLQLKPNITNSARHLLEGLLQKDRTKRLGAKDDFMEIKSHVFFSLINWDDLINKKITPPFNPNVSGPNDLRHFDPEFTEEPVPNSIGKSPDSVLVTASVKEAAEAFLGFSYAPPTDSFL

>NP_001137149.1 serine/threonine-protein kinase Sgk1 isoform 3 [Homo sapiens]

MSSQSSSLSEACSREAYSSHNWALPPASRSNPQPAYPWATRRMKEEAIKPPLKAFMKQRRMGLNDFIQKIANNSYACKHPEVQSILKISQPQEPELMNANPSPPPSPSQQINLGPSSNPHAKPSDFHFLKVIGKGSFGKVLLARHKAEEVFYAVKVLQKKAILKKKEEKHIMSERNVLLKNVKHPFLVGLHFSFQTADKLYFVLDYINGGELFYHLQRERCFLEPRARFYAAEIASALGYLHSLNIVYRDLKPENILLDSQGHIVLTDFGLCKENIEHNSTTSTFCGTPEYLAPEVLHKQPYDRTVDWWCLGAVLYEMLYGLPPFYSRNTAEMYDNILNKPLQLKPNITNSARHLLEGLLQKDRTKRLGAKDDFMEIKSHVFFSLINWDDLINKKITPPFNPNVSGPNDLRHFDPEFTEEPVPNSIGKSPDSVLVTASVKEAAEAFLGFSYAPPTDSFL

>NP_001137150.1 serine/threonine-protein kinase Sgk1 isoform 4 [Homo sapiens]

MGEMQGALARARLESLLRPRHKKRAEAQKRSESFLLSGLAFMKQRRMGLNDFIQKIANNSYACKHPEVQSILKISQPQEPELMNANPSPPPSPSQQINLGPSSNPHAKPSDFHFLKVIGKGSFGKVLLARHKAEEVFYAVKVLQKKAILKKKEEKHIMSERNVLLKNVKHPFLVGLHFSFQTADKLYFVLDYINGGELFYHLQRERCFLEPRARFYAAEIASALGYLHSLNIVYRDLKPENILLDSQGHIVLTDFGLCKENIEHNSTTSTFCGTPEYLAPEVLHKQPYDRTVDWWCLGAVLYEMLYGLPPFYSRNTAEMYDNILNKPLQLKPNITNSARHLLEGLLQKDRTKRLGAKDDFMEIKSHVFFSLINWDDLINKKITPPFNPNVSGPNDLRHFDPEFTEEPVPNSIGKSPDSVLVTASVKEAAEAFLGFSYAPPTDSFL

>NP_001278924.1 serine/threonine-protein kinase Sgk1 isoform 5 [Homo sapiens]

MTVKTEAAKGTLTYSRMRGMVAILIAFMKQRRMGLNDFIQKIANNSYACKHPEVQSILKISQPQEPELMNANPSPPPSPSQQINLGPSSNPHAKPSDFHFLKVIGKGSFGKVLLARHKAEEVFYAVKVLQKKAILKKKELFYHLQRERCFLEPRARFYAAEIASALGYLHSLNIVYRDLKPENILLDSQGHIVLTDFGLCKENIEHNSTTSTFCGTPEYLAPEVLHKQPYDRTVDWWCLGAVLYEMLYGLPPFYSRNTAEMYDNILNKPLQLKPNITNSARHLLEGLLQKDRTKRLGAKDDFMEIKSHVFFSLINWDDLINKKITPPFNPNVSGPNDLRHFDPEFTEEPVPNSIGKSPDSVLVTASVKEAAEAFLGFSYAPPTDSFL

**CLUSTAL O(1.2.4) multiple sequence alignment of human SGK1 protein sequences**

2 MVNKDMNGFPVKKCSAFQFFKKRVRRWIKSPMVSVDKHQSPSLKYTGSSMVHIPPGEPDF 60

4 --------------------------------------------------------MGEM 4

3 --------------------------------------------------------MSSQ 4

1 ------------------------------------------------------------ 0

5 ------------------------------------------------------------ 0

2 ESSLCQTCLGEHAFQRGVLPQENESCSWETQSGCEVREPCNHANILTKPDPRTF------ 114

4 QGALA----------------------RARL---ESLLR---PRHK-------KRAEAQK 29

3 SSSLSE------------------ACSREAYSSHNWALP---PASRSNPQPAYPWATRRM 43

1 -------------------------------------MT----VKTEAAKGTLT----YS 15

5 -------------------------------------MT----VKTEAAKGTLT----YS 15

2 ----WTNDDPAFMKQRRMGLNDFIQKIANNSYACKHPEVQSILKISQPQEPELMNANPSP 170

4 RSESFLLSGLAFMKQRRMGLNDFIQKIANNSYACKHPEVQSILKISQPQEPELMNANPSP 89

3 KEEAIKPPLKAFMKQRRMGLNDFIQKIANNSYACKHPEVQSILKISQPQEPELMNANPSP 103

1 RMRGMVAILIAFMKQRRMGLNDFIQKIANNSYACKHPEVQSILKISQPQEPELMNANPSP 75

5 RMRGMVAILIAFMKQRRMGLNDFIQKIANNSYACKHPEVQSILKISQPQEPELMNANPSP 75

**************************************************

2 PPSPSQQINLGPSSNPHAKPSDFHFLKVIGKGSFGKVLLARHKAEEVFYAVKVLQKKAIL 230

4 PPSPSQQINLGPSSNPHAKPSDFHFLKVIGKGSFGKVLLARHKAEEVFYAVKVLQKKAIL 149

3 PPSPSQQINLGPSSNPHAKPSDFHFLKVIGKGSFGKVLLARHKAEEVFYAVKVLQKKAIL 163

1 PPSPSQQINLGPSSNPHAKPSDFHFLKVIGKGSFGKVLLARHKAEEVFYAVKVLQKKAIL 135

5 PPSPSQQINLGPSSNPHAKPSDFHFLKVIGKGSFGKVLLARHKAEEVFYAVKVLQKKAIL 135

************************************************************

2 KKKEEKHIMSERNVLLKNVKHPFLVGLHFSFQTADKLYFVLDYINGGELFYHLQRERCFL 290

4 KKKEEKHIMSERNVLLKNVKHPFLVGLHFSFQTADKLYFVLDYINGGELFYHLQRERCFL 209

3 KKKEEKHIMSERNVLLKNVKHPFLVGLHFSFQTADKLYFVLDYINGGELFYHLQRERCFL 223

1 KKKEEKHIMSERNVLLKNVKHPFLVGLHFSFQTADKLYFVLDYINGGELFYHLQRERCFL 195

5 KKK--------------------------------------------ELFYHLQRERCFL 151

*** *************

2 EPRARFYAAEIASALGYLHSLNIVYRDLKPENILLDSQGHIVLTDFGLCKENIEHNSTTS 350

4 EPRARFYAAEIASALGYLHSLNIVYRDLKPENILLDSQGHIVLTDFGLCKENIEHNSTTS 269

3 EPRARFYAAEIASALGYLHSLNIVYRDLKPENILLDSQGHIVLTDFGLCKENIEHNSTTS 283

1 EPRARFYAAEIASALGYLHSLNIVYRDLKPENILLDSQGHIVLTDFGLCKENIEHNSTTS 255

5 EPRARFYAAEIASALGYLHSLNIVYRDLKPENILLDSQGHIVLTDFGLCKENIEHNSTTS 211

************************************************************

2 TFCGTPEYLAPEVLHKQPYDRTVDWWCLGAVLYEMLYGLPPFYSRNTAEMYDNILNKPLQ 410

4 TFCGTPEYLAPEVLHKQPYDRTVDWWCLGAVLYEMLYGLPPFYSRNTAEMYDNILNKPLQ 329

3 TFCGTPEYLAPEVLHKQPYDRTVDWWCLGAVLYEMLYGLPPFYSRNTAEMYDNILNKPLQ 343

1 TFCGTPEYLAPEVLHKQPYDRTVDWWCLGAVLYEMLYGLPPFYSRNTAEMYDNILNKPLQ 315

5 TFCGTPEYLAPEVLHKQPYDRTVDWWCLGAVLYEMLYGLPPFYSRNTAEMYDNILNKPLQ 271

************************************************************

2 LKPNITNSARHLLEGLLQKDRTKRLGAKDDFMEIKSHVFFSLINWDDLINKKITPPFNPN 470

4 LKPNITNSARHLLEGLLQKDRTKRLGAKDDFMEIKSHVFFSLINWDDLINKKITPPFNPN 389

3 LKPNITNSARHLLEGLLQKDRTKRLGAKDDFMEIKSHVFFSLINWDDLINKKITPPFNPN 403

1 LKPNITNSARHLLEGLLQKDRTKRLGAKDDFMEIKSHVFFSLINWDDLINKKITPPFNPN 375

5 LKPNITNSARHLLEGLLQKDRTKRLGAKDDFMEIKSHVFFSLINWDDLINKKITPPFNPN 331

************************************************************

2 VSGPNDLRHFDPEFTEEPVPNSIGKSPDSVLVTASVKEAAEAFLGFSYAPPTDSFL 526

4 VSGPNDLRHFDPEFTEEPVPNSIGKSPDSVLVTASVKEAAEAFLGFSYAPPTDSFL 445

3 VSGPNDLRHFDPEFTEEPVPNSIGKSPDSVLVTASVKEAAEAFLGFSYAPPTDSFL 459

1 VSGPNDLRHFDPEFTEEPVPNSIGKSPDSVLVTASVKEAAEAFLGFSYAPPTDSFL 431

5 VSGPNDLRHFDPEFTEEPVPNSIGKSPDSVLVTASVKEAAEAFLGFSYAPPTDSFL 387

********************************************************

**SGK1F forms**

>CAR58098.1 serum/glucocorticoid regulated kinase 1 variant F [Homo sapiens]

MKPSKRFFISPPSSTAFMKQRRMGLNDFIQKIANNSYACKHPEVQSILKISQPQEPELMNANPSPPPSPSQQINLGPSSNPHAKPSDFHFLKVIGKGSFGKVLLARHKAEEVFYAVKVLQKKAILKKKEEKHIMSERNVLLKNVKHPFLVGLHFSFQTADKLYFVLDYINGGELFYHLQRERCFLEPRARFYAAEIASALGYLHSLNIVYRDLKPENILLDSQGHIVLTDFGLCKENIEHNSTTSTFCGTPEYLAPEVLHKQPYDRTVDWWCLGAVLYEMLYGLPPFYSRNTAEMYDNILNKPLQLKPNITNSARHLLEGLLQKDRTKRLGAKDDFMEIKSHVFFSLINWDDLINKKITPPFNPNVSGPNDLRHFDPEFTEEPVPNSIGKSPDSVLVTASVKEAAEAFLGFSYAPPTDSFL

>AdV-encoded_SGK1F

MDYKDDDDKKPSKRFFISPPSSTAFMKQRRMGLNDFIQKIANNSYACKHPEVQSILKISQPQEPELMNANPSPPPSPSQQINLGPSSNPHAKPSDFHFLKVIGKGSFGKVLLARHKAEEVFYAVKVLQKKAILKKKEEKHIMSERNVLLKNVKHPFLVGLHFSFQTADKLYFVLDYINGGELFYHLQRERCFLEPRARFYAAEIASALGYLHSLNIVYRDLKPENILLDSQGHIVLTDFGLCKENIEHNSTTSTFCGTPEYLAPEVLHKQPYDRTVDWWCLGAVLYEMLYGLPPFYSRNTAEMYDNILNKPLQLKPNITNSARHLLEGLLQKDRTKRLGAKDDFMEIKSHVFFSLINWDDLINKKITPPFNPNVSGPNDLRHFDPEFTEEPVPNSIGKSPDSVLVTASVKEAAEAFLGFSYAPPTDSFL

**CLUSTAL O(1.2.4) multiple sequence alignment of SGK1F (F) with human SGK1 isoform 1 (1)**

F ----------MKPSKRFFISPPSSTAFMKQRRMGLNDFIQKIANNSYACKHPEVQSILKI 50

1 MTVKTEAAKGTLTYSRMRGMVAILIAFMKQRRMGLNDFIQKIANNSYACKHPEVQSILKI 60

.*: ***********************************

F SQPQEPELMNANPSPPPSPSQQINLGPSSNPHAKPSDFHFLKVIGKGSFGKVLLARHKAE 110

1 SQPQEPELMNANPSPPPSPSQQINLGPSSNPHAKPSDFHFLKVIGKGSFGKVLLARHKAE 120

************************************************************

F EVFYAVKVLQKKAILKKKEEKHIMSERNVLLKNVKHPFLVGLHFSFQTADKLYFVLDYIN 170

1 EVFYAVKVLQKKAILKKKEEKHIMSERNVLLKNVKHPFLVGLHFSFQTADKLYFVLDYIN 180

************************************************************

F GGELFYHLQRERCFLEPRARFYAAEIASALGYLHSLNIVYRDLKPENILLDSQGHIVLTD 230

1 GGELFYHLQRERCFLEPRARFYAAEIASALGYLHSLNIVYRDLKPENILLDSQGHIVLTD 240

************************************************************

F FGLCKENIEHNSTTSTFCGTPEYLAPEVLHKQPYDRTVDWWCLGAVLYEMLYGLPPFYSR 290

1 FGLCKENIEHNSTTSTFCGTPEYLAPEVLHKQPYDRTVDWWCLGAVLYEMLYGLPPFYSR 300

************************************************************

F NTAEMYDNILNKPLQLKPNITNSARHLLEGLLQKDRTKRLGAKDDFMEIKSHVFFSLINW 350

1 NTAEMYDNILNKPLQLKPNITNSARHLLEGLLQKDRTKRLGAKDDFMEIKSHVFFSLINW 360

************************************************************

F DDLINKKITPPFNPNVSGPNDLRHFDPEFTEEPVPNSIGKSPDSVLVTASVKEAAEAFLG 410

1 DDLINKKITPPFNPNVSGPNDLRHFDPEFTEEPVPNSIGKSPDSVLVTASVKEAAEAFLG 420

************************************************************

F FSYAPPTDSFL 421

1 FSYAPPTDSFL 431

***********
